# Mometasone furoate in the treatment of mild, moderate, or severe persistent allergic rhinitis: a non-inferiority study (PUMA)^☆^

**DOI:** 10.1016/j.bjorl.2015.11.009

**Published:** 2016-02-15

**Authors:** Martti Anton Antila, Fabio Morato Castro, Flavio Sano, Adelmir Machado, Fatima Fernandes, Nelson Augusto Rosário Filho, Rafael Stelmach

**Affiliations:** a Clínica de Alergia Martti Antila (CMPC), Sorocaba, SP, Brazil; b Universidade de São Paulo (FMUSP), Faculdade de Medicina, São Paulo, SP, Brazil; c Hospital Nipo-brasileiro, São Paulo, SP, Brazil; d Universidade Federal da Bahia (UFBA), Instituto de Ciências da Saúde, Salvador, BA, Brazil; e Fundação José Luiz Egydio Setúbal (Hospital Infantil Sabará), São Paulo, SP, Brazil; f Universidade Federal do Paraná (UFPR), Departamento de Pediatria, Curitiba, PR, Brazil

**Keywords:** Persistent allergic rhinitis, Mometasone furoate, Non-inferiority, Rinite alérgica persistente, Furoato de mometasona, Não inferioridade

## Abstract

**Introduction:**

Allergic rhinitis is considered the most prevalent respiratory disease in Brazil and worldwide, with great impact on quality of life, affecting social life, sleep, and also performance at school and at work.

**Objective:**

To compare the efficacy and safety of two formulations containing mometasone furoate in the treatment of mild, moderate, or severe persistent allergic rhinitis after four weeks of treatment.

**Methods:**

Phase III, randomized, non-inferiority, national, open study comparing mometasone furoate in two presentations (control drug and investigational drug). The primary endpoint was the percentage of patients with reduction of at least 0.55 in nasal index score (NIS) after four weeks of treatment. Secondary outcomes included total nasal index score score after four and 12 weeks of treatment; individual scores for symptoms of nasal obstruction, rhinorrhea, sneezing, and nasal pruritus; as well as score for pruritus, lacrimation, and ocular redness after four and 12 weeks of treatment. The study was registered at clinicaltrials.gov with the reference number NCT01372865.

**Results:**

The efficacy primary analysis demonstrated non-inferiority of the investigational drug in relation to the control drug, since the upper limit of the confidence interval (CI) of 95% for the difference between the success rates after four weeks of treatment (12.6%) was below the non-inferiority margin provided during the determination of the sample size (13.7%). Adverse events were infrequent and with mild intensity in most cases.

**Conclusion:**

The efficacy and safety of investigational drug in the treatment of persistent allergic rhinitis were similar to the reference product, demonstrating its non-inferiority.

## Resumo

**Introdução:**

A rinite alérgica é considerada a doença respiratória mais prevalente no Brasil e em todo o mundo, com grande impacto na qualidade de vida; além de, afetar a vida social, o sono e também o desempenho na escola e no trabalho.

**Objetivo:**

Comparar a eficácia e segurança de duas formulações contendo furoato de mometasona no tratamento da rinite alérgica persistente leve, moderada ou grave por um período de quatro semanas.

**Método:**

Trata-se de um estudo nacional aberto de fase III, randomizado, de não inferioridade de comparação do furoato de mometasona em duas apresentações (medicação de controle e fármaco sob investigação). O ponto final primário foi o percentual de pacientes com redução mínima de 0,55 no escore de índice nasal (EIN) após quatro semanas de tratamento. Os desfechos secundários foram: escore NIS total após 4 e 12 semanas de tratamento; escores individuais para sintomas de obstrução nasal, rinorréia, espirros e prurido nasal, bem como escores para prurido, lacrimejamento e hiperemia conjuntival após 4 e 12 semanas de tratamento. O estudo foi registrado em clinicaltrials.gov com o número de referência NCT01372865.

**Resultados:**

A análise de eficácia primária demonstrou não inferioridade do fármaco sob investigação em relação à medicação de controle, visto que o limite superior do intervalo de confiança (IC) de 95% para a diferença entre os percentuais de sucesso após quatro semanas de tratamento (12,6%) situava-se abaixo da margem de não inferioridade proporcionada durante a determinação do tamanho da amostra (13,7%). Eventos adversos foram pouco frequentes e de leve intensidade na maioria dos casos.

**Conclusão:**

A eficácia e a segurança de um fármaco experimental no tratamento da rinite alérgica persistente foram similares às do produto de referência, o que demonstrou sua não inferioridade.

## Introduction

Allergic rhinitis is an allergic disease characterized by chronic inflammation of the mucous membranes of the respiratory tract. Its main symptoms are nasal congestion, sneezing, anterior and posterior rhinorrhea, nasal pruritus, ocular and palatal pruritus, conjunctival injection, and lacrimation.^1^, ^2^

Considered the most prevalent respiratory disease in Brazil and worldwide, recent estimates indicate that approximately 500 million people suffer from allergic rhinitis.^1^, ^3^ In the United States, it is believed that 10–30% of adults and 40% of children are affected by allergic rhinitis.^4^ In Brazil, it is estimated that the average prevalence in adolescents and schoolchildren is 29.6% and 25.7%, respectively.^3^, ^5^

Clinical manifestations of allergic rhinitis occur after the interaction of a specific allergen and the immune system of previously sensitized individual. Immediate hypersensitivity is a fast reaction with participation of IgE and mast cells followed by inflammation.^6^ Other allergic diseases may be associated with rhinitis, such as asthma and atopic dermatitis.^7^

In 1999, the Allergic Rhinitis and its Impact on Asthma (ARIA) workgroup,^1^, ^8^ in collaboration with World Health Organization (WHO), provided a base of evidence to facilitate the diagnosis and treatment of the disease. The group recommended that allergic rhinitis be classified according to the duration (intermittent or persistent) and intensity of symptoms (mild or moderate/severe).^9^

Although benign, allergic rhinitis has a significant impact on quality of life, affecting social life, sleep, as well as school and labor performance.^1^, ^8^ The main goal of treatment is to prevent or relieve symptoms with maximum safety and efficacy. Ideally, the treatment of allergic rhinitis should aim primarily the action on cells and inflammatory mediators, thereby minimizing the symptoms of the disease. Several classes of drugs are used in the treatment, such as oral or topical antihistamines, intranasal decongestants, leukotriene receptor antagonists, and topical intranasal corticosteroids. The latter are recognized as the first-choice drugs in anti-inflammatory treatment of moderate to severe allergic diseases.^1^, ^8^, ^10^, ^11^

The mechanism of action of corticosteroids includes chemical mediators and cells involved in the allergic inflammatory process that establishes the rhinitis. Intranasal formulations have the advantage of local administration, with faster onset of action compared to systemic therapies. Moreover, it has been reported that corticosteroids may help control comorbidities of rhinitis, such as allergic conjunctivitis and asthma.^10^, ^12^

Mometasone furoate is a synthetic glucocorticoids for topical intranasal use,^13^ which inhibits the formation, release, and activity of endogenous chemical mediators, also limiting cellular phase of allergic inflammation. Its intranasal application controls the initial and late allergic response.^14^

Given the previously demonstrated efficacy of mometasone furoate in the treatment of allergic rhinitis, the present study was designed to test the non-inferiority of the new formulation compared to the control product. The primary objective was to compare the efficacy of both drugs in the treatment of persistent allergic rhinitis after four weeks.

## Methods

This was a phase III, randomized, non-inferiority, national, multicenter (seven centers), open study, aiming to compare the new experimental formulation of mometasone furoate (Eurofarma) to the control drug (Nasonex^®^, Schering-Plough Pharmaceuticals Ltd.) administered in total daily dose of 200 μg (two sprays of 100 μg in each nostril once daily).

The study was approved by the research ethics committees of the institutions involved under the number CE No. 106/2012 (issued on May 17th, 2012) and all study participants signed an informed consent (IC). In addition, it was sponsored by Eurofarma Laboratórios S.A. Patients with mild, moderate, or severe persistent allergic rhinitis were included, according to the ARIA criteria,^1^, ^8^ with total nasal index score (NIS) value ≥4 at the screening visit and on at least four of the seven days preceding this visit. Patients also were required to be >12 years of age, with symptoms of allergic rhinitis for at least two years (confirmed by a positive skin test to at least one relevant aero-allergen conducted over the past 90 days), and also have prescribed use of nasal corticosteroid. Furthermore, patients were required to undergo a washout period of 14 ± 5 days between the screening (SV) and the randomization visit (RV) without use of any nasal, oral, or parenteral corticosteroids (including antihistamines; oral or topical nasal vasoconstrictor; and corticosteroids in any route of administration – except cutaneous). If the research subject required other medication, he/she was excluded from the study to continue treatment according to local practices.

The study was conducted in open-label setting, with no blinding of interventions. Blinding prevents possible biases in clinical trials; however, sometimes it cannot be applied. This study was open because the drugs studied have very different appearances, which would make the blinding infeasible. Nevertheless, the symptoms diary and the additional tests prevented the occurrence of any bias in the evaluation of the researchers. Thus, the fact that this study was open does not impact the quality of data collected.

Patients with severe comorbidities (according to the investigator criteria); moderate to severe persistent asthma; history of respiratory tract infection within 30 days prior to the study entry; structural changes causing nasal obstruction (excessively deviated septum, nasal polyps, or any type of nasal malformation); as well as patients in need of other medicines to treat allergic rhinitis; pregnant or lactating patients; active smokers in the last three months prior to enrollment in the study; and those who participated in another clinical study in the past 12 months were not included in the study.

After stratification according to the research center and to the intensity of allergic rhinitis (mild versus moderate or severe), patients were randomized in a 1:1 ratio to receive one of the study treatments. The research subjects were randomized centrally, according to a list created by an application to generate random sequences. The randomization and allocation of treatment was conducted electronically through an electronic case report file (CRF). Treatment was automatically registered in the appropriate field on the medical records of the study. All randomized patients received at least one dose of study medication and received no different drug than that he or she was randomized to receive. The follow-up period for each patient was 14 weeks and the scheduled duration of active treatment was 12 weeks.

After RV, patients were assessed in four visits (V1, V2, V3, and FV). During the study, patients completed a diary containing information about the symptoms of allergic rhinitis, nasal obstruction, rhinorrhea, sneezing and nasal pruritus, as well as the score for pruritus, lacrimation, and ocular redness after four and 12 weeks of treatment, drug adherence, and use of rescue medications. At each visit the symptoms were rated on a scale from 0 to 3, which was summed to constitute the endpoint of NIS in the study period.

The primary endpoint was the proportion of patients with a reduction of at least 0.55 points in the NIS score after four weeks of treatment, compared with baseline, using data recorded in the diary. This threshold was defined based on the study conducted by Barnes et al.,^15^ in which it was determined that a reduction of 0.55 points in another combined score, the total nasal symptom score (TNSS), is the minimal clinically important difference (MCID) and, therefore, can be considered clinically significant for patients treated with intranasal corticosteroids. Similar to the NIS, the TNSS is the sum of scores for a group of symptoms, each measured on an ordinal scale of 0, 1, 2, or 3, representing no symptoms, mild, moderate, or severe symptoms, respectively. Instead of 3, the TNSS combines the score of four symptoms (nasal run, blockage, itchiness, and sneeze), so it can range from 0 to 12. As there was no such data on the MCID for the NIS, and in both scores, symptoms are individually scored from 0 to 3 and contribute equally to the total sum (no different weight is attributed to different symptoms), it was assumed that if a 0.55 reduction is clinically significant in a scale from 0 to 12 it would also be in a 0 to 9 scale. If a proportional extrapolation of the threshold was done, a reduction of 0.55 in the 0 to 12 point scale (TNSS) would represent a reduction of 0.41 in the 0 to 9 NIS scale. Thus, an approach using the threshold of 0.55 point in the NIS to define clinical benefit can be considered conservative.

Secondary outcomes included total NIS score after four and 12 weeks of treatment; assessment of individual and overall scores for symptoms of nasal obstruction, rhinorrhea, and sneezing using NIS score; and assessment of scores of nasal pruritus and pruritus, lacrimation, and ocular redness performed by the investigator at each visit to the research center; as well as the frequency of adverse events.

### Statistical methods

To calculate NIS at baseline, the mean values of valid measures of the score for each patient in the seven days preceding the RV were used. To calculate NIS after four weeks, the mean values of the fourth week of treatment were used, considering the data recorded in the diary.

In the present study, the main efficacy measure was the NIS, a combined score calculated by the sum of individual scores of three nasal symptoms (nasal congestion/obstruction, runny nose, and sneezing). Each symptom was individually graded from 0 (no symptom) to 3 (severe symptoms), and the total NIS can range from 0 to 9. Although the TNSS score is more frequently used, the NIS is a valid scale and has been used in several studies to assess the efficacy of intranasal corticosteroids in seasonal and perennial rhinitis.^16^, ^17^, ^18^, ^19^

For the per protocol (PP) population, the results obtained for nasal congestion (obstruction), runny nose, and sneezing are shown as individual symptoms and as a combined score (NIS). In addition to these three symptoms that constitute the NIS, patients had to grade in their diaries the other two symptoms (one: nasal pruritus, and two: pruritus, lacrimation, and ocular redness), which were analyzed individually.

No imputation of missing data was taken. Continuous variables were summarized by means of variation (minimum and maximum), mean, standard deviation (SD), median, and interquartile range (IQR, 25th percentile, and 75th percentile). Categorical variables were described by means of relative frequencies.

When comparing both groups, parametric or non-parametric methods were used, according to the distribution pattern of the outcome variables. The Kolmogorov–Smirnov test with Lilliefors correction was used to assess the pattern of distribution of the outcome variables in the sample.

Continuous variables with normal distribution were compared using *t*-tests, while non-normally distributed variables were compared using the Mann–Whitney non-parametric test. Categorical variables were compared using the chi-squared or Fisher's exact test, according to the number of individuals. For the multivariate analysis of the proportions of patients with reduction of ≥0.55 in NIS total score over time in both groups, the ANOVA test with repeated measures was used, with Greenhouse–Geisser correction.

Two-tailed levels of significance of 5% were used as indicative of statistical differences between groups. For declaration of non-inferiority of the investigational drug compared to the control drug, the upper limit of the 95% CI for the difference between the proportions of patients in both groups with reduction of ≥0.55 in the total NIS score (control drug *minus* investigational drug) after four weeks of treatment should be up to 13.7% in absolute values. If non-inferiority was demonstrated, a superiority test would be performed, with a two-tailed level of 5% as indicative of significant difference between groups.

Analyses were performed using Microsoft Excel software (Microsoft Office 2007) for descriptive analyses, and R statistical software (v. 2.13.1) and MedCalc (v. 11.3.3.0, Mariakerke, Belgium), for inferential analyzes.

### Sample size determination

Based on the literature, the determination of sample size in the non-inferiority design of the study considered the NIS score after four weeks of equal treatment in both groups. Considering a one-tailed alpha error of 2.5% and a statistical power of 80% for the study to find the maximum difference of 13.7% or less between the control and investigational arms (limit for non-inferiority), 164 patients would be included in each arm of the study. Assuming that there would be a loss of follow up of approximately 15%, the study would include 193 patients in each arm, i.e., a total of 386 patients.

A non-inferiority margin (M) of 13.7% was determined. The determination of the non-inferiority margin was based on the maximum acceptable difference between experimental and control groups, as judged by the specialists, in order to assure for the experimental group the retention of a minimum of the beneficial effect of treatment in relation to placebo or absence of medication. In addition, the non-inferiority margin chosen for this study is in accordance with the *European Medicines Agency*'s guidelines and other publications that recommend the use of margins between 10% and 20%, depending on the therapeutic field.

## Results

### Study population

Between May and September 2012, 568 subjects with mild, moderate, or severe persistent allergic rhinitis, classified according to the ARIA criteria, were included in the study, and 387 of them were randomized to receive one of the study drugs. The reasons for randomization failure are shown in Table 1.

**Table 1:** Reasons for randomization failure.

Reason	*n* (%)
Eligibility failure	105 (58.2)
Loss to follow-up	4 (2.2)
Consent withdrawal	9 (4.8)
Lack of adherence to protocol	6 (3.3)
Use of medications not allowed in the study	11 (6.1)
End of position in one of the strata in the study	46 (25.4)

**Figure 1:**
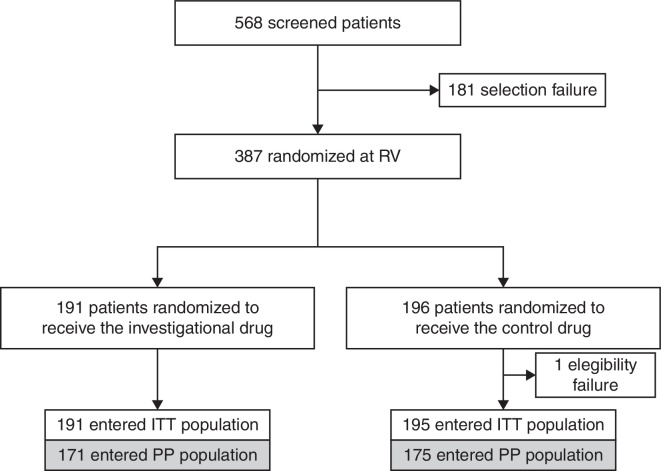
Flow of patients in the study (ITT, intent to treat population; PP, per-protocol population; RV, randomization visit).

All subjects enrolled received at least one dose of the study medication and none had received a different drug than the one established. Fig. 1 shows the patients flow in the study according to the treatment groups.

All of the 387 randomized patients received at least one dose of the study medication and entered the safety sample. One randomized subject violated an eligibility criterion and the study was discontinued at the time the violation became known. Thus, this subject was excluded from the intention-to-treat (ITT) and per protocol (PP) subgroups.

Forty of the 386 subjects in the ITT population did not enter the PP population due to: lack of data for evaluation of the primary endpoint (*n* = 23); use of medication not allowed in the study (*n* = 10); or use of higher doses of rescue medication between week one and week four of the study (*n* = 7). The ITT population was therefore composed of 386 patients, the PP population of 346 patients, and the safety population of 386 patients.

Seventy-seven of 386 randomized patients were prematurely discontinued from the study due to concomitant disease (*n* = 4), lack of adherence to protocol or treatment (*n* = 19), loss to follow up (*n* = 24), consent withdrawal (*n* = 3), toxicity or adverse events (*n* = 2) or use of medications not allowed in the protocol during the study (*n* = 24).

The demographic characteristics of patients are presented in Table 2.

**Table 2:** Demographic characteristics of the ITT and PP subgroups.

Characteristic	ITT population (*n* = 386)		PP Population (*n* = 346)	
	Investigational drug (*n* = 191)	Control drug (*n* = 195)	Investigational drug (*n* = 171)	Control drug (*n* = 175)
*Gender, n (%)*				
Women	119 (62.30)	117 (60.00)	107 (62.57)	108 (61.71)
Men	72 (37.70)	78 (40.00)	64 (37.43)	67 (38.29)
*Age, years (mean* *±* *SD)*	27.74 ± 12.51	31.04 ± 14.41	28.04 ± 12.81	31.15 ± 14.42
*Range*	12.19–70.38	12.01–67.54	12.19–70.38	12.00–67.54
*Median (IQR)*	25.12 (17.60–34.39)	28.79 (18.68–41.35)	25.39 (17.69–34.94)	28.79 (19.01–41.40)

*Ethnicity, n (%)*				
White	112 (58.64)	120 (61.54)	99 (57.89)	109 (62.29)
Mixed	32 (16.75)	33 (16.92)	28 (16.37)	27 (15.43)
African-American	42 (21.99)	37 (18.97)	39 (22.81)	34 (19.43)
Asian	5 (2.62)	5 (2.56)	5 (2.92)	5 (2.86)
*BMI, km/m*^*2*^*(mean* *±* *SD)*	24.96 ± 5.39	25.02 ± 5.07	24.65 ± 5.36	25.03 ± 4.84
*Range*	15.27–44.31	15.27–45.92	15.27–44.31	15.27–39.81
*Median (IQR)*	24.13 (20.76–28.41)	24.61 (21.90–27.29)	23.72 (20.55–28.13)	24.64 (21.95–27.36)

*History of smoking; n (%)*				
Non-smokers	177 (92.67)	181 (92.82)	158 (92.40)	162 (92.57)
Ex-smokers^a^	14 (7.33)	14 (7.18)	13 (7.60)	13 (7.43)

SD, standard deviation; BMI, body mass index; NIS, nasal index score; IQR, interquartile range; ITT, intent to treat population; PP, per-protocol population.

a Stopped smoking three months prior to study entry.

### Efficacy endpoint

**Figure 2:**
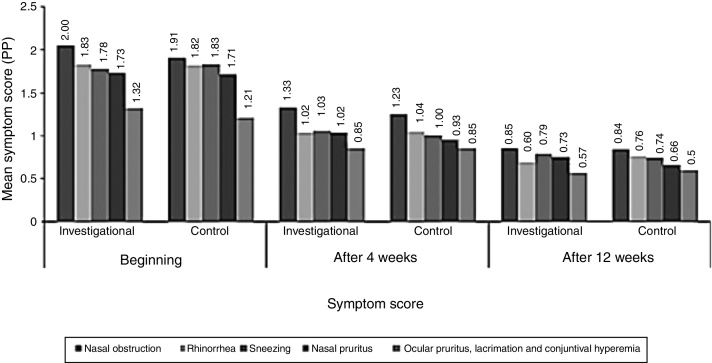
Symptom score.

Symptoms of allergic rhinitis were evaluated by NIS score, which included the evaluation of individual symptoms such as nasal congestion/obstruction, runny nose, and sneezing. In addition to these symptoms, patients had to grade in their diaries the other two symptoms: nasal pruritus; and pruritus, lacrimation, and ocular redness; which were analyzed individually. There was no significant difference between the groups, except for nasal obstruction: according to the data recorded in the diary, the score for nasal obstruction at baseline was significantly higher in the investigational arm than in the control arm (Fig. 2).

The efficacy primary analysis was performed by evaluating the non-inferiority of the investigational drug compared to the control drug. Table 3 shows the comparison between the success rates in the two treatment groups in PP population. It can be observed that 76.6% of patients in the investigational arm and 80% in the control arm had a reduction of at least 0.55 in NIS score after four weeks of treatment and that there was no statistically significant difference between groups (*p* = 0.586).

**Table 3:** Comparison of the proportions of patients with a reduction of at least 0.55 in NIS score after four weeks of treatment in both groups, PP population (*n* = 346).

Reduction of at least 0.55 in NIS score after 4 weeks of treatment	Investigational drug, *n* (%)	Control drug, *n* (%)	*p*^a^
	(*n* = 171)	(*n* = 175)	
Yes	131 (76.6)	140 (80.0)	0.586
No	40 (23.4)	35 (20.0)	

PP, per-protocol population; NIS, nasal index score.

a Mann–Whitney test.

**Figure 3:**
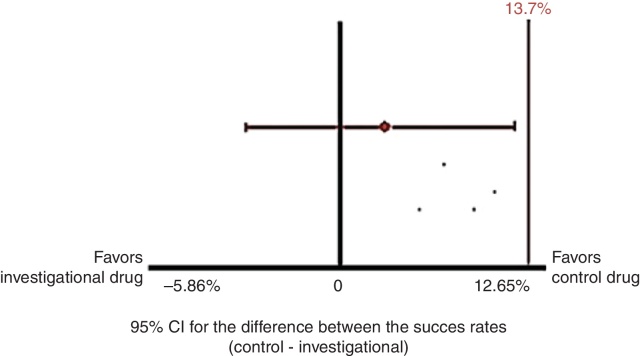
Primary analysis of efficacy in the PP population (*n* = 346).

The 95% CI for the difference between the success rates (investigational drug and control drug) after four weeks of treatment, obtained by proportions equality test with continuity correction, was −5.87 to 12.65. Fig. 3 shows the results graphically, with the upper limit of the CI (12.7%) below the non-inferiority margin (*M* = 13.7%). Thus, the non-inferiority of the investigational drug compared to the control drug was demonstrated.

**Figure 4:**
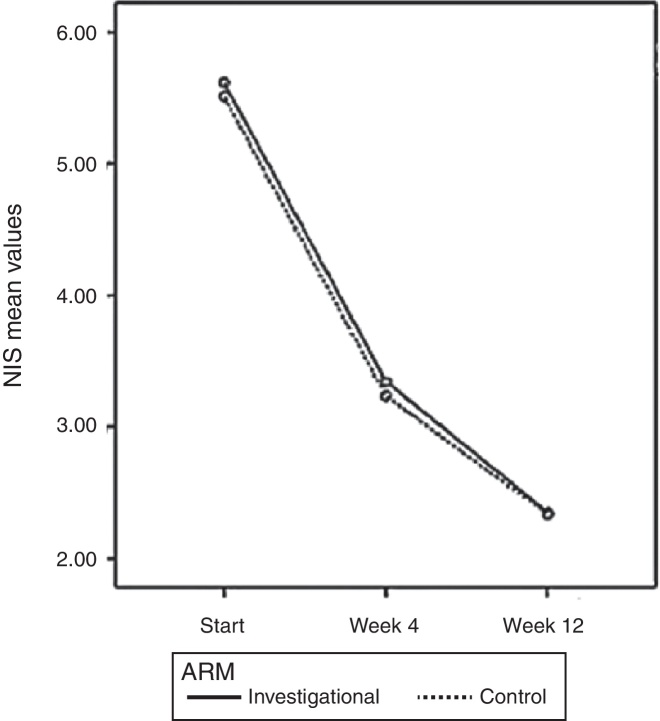
Total NIS score over time based on patient diary, PP population (*n* = 308).

As observed in the PP population, there was a significant effect of treatment time on the values of NIS (*p* < 0.0001) and no significant difference between groups (*p* = 0.624) in the ITT population. The values of NIS score in the investigational arm were 5.61 ± 1.52; 3.34 ± 2.06; and 2.34 ± 1.91 before treatment and after four and 12 weeks of treatment, respectively. The group treated with the control drug showed a NIS score of 5.51 ± 1.36; 3.24 ± 2.07; and 2.34 ± 1.91 before treatment and after four and 12 weeks of treatment, respectively (Fig. 4).

### Use of rescue medication

After the beginning of the treatment, 54.3% of the patients who received the control drug and 45.71% of those receiving the investigational drug used ebastine during the study treatment. There was no significant difference between the treatment arms.

### Safety results

Table 4 shows the level of serum cortisol in the beginning of the study (SV) and after four weeks of treatment (FV) in both treatment groups, considering the safety population. The comparison of medians showed no significant difference between groups in the beginning or in the end of treatment.

**Table 4:** Level of serum cortisol in SV and FV (mg/dL) in the safety population (*n* = 387).

Cortisol	Investigational drug	Control drug	*p*^a^
SV, μg/dL	*n* = 191	*n* = 196	
Range	0.29–40.11	2.26–38.51	
Mean ± SD	11.97 ± 7.27	11.91 ± 6.52	
Median (IQR)	10.30 (6.70–15.40)	10.44 (7.34–15.20)	0.736

FV, μg/dL	*n* = 168	*n* = 171	
Range	2.90–37.87	1.0–39.48	
Mean ± SD	13.64 ± 7.05	13.17 ± 6.60	
Median (IQR)	12.10 (8.84–17.01)	11.90 (8.70–15.91)	0.747

SD, standard deviation; IQR, interquartile range; SV, screening visit; FV, final visit.

a Mann–Whitney test.

In the safety population, 235 patients presented non-serious adverse events during the study. In the control group, 119 of 191 patients (62.30%) had an adverse event, and in the investigational arm, 116 of 196 patients (59.2%) presented an adverse event (*p* = 0.530). With the exception of epistaxis and myalgia, more frequent in the control arm, and abnormal electrocardiogram, more frequent in the investigational arm, there was no significant difference between groups in the frequency of adverse events. The most frequent adverse events in both groups were back pain, cough, dysmenorrhea, headache, and nasopharyngitis.

There was a report of a single serious adverse event during the study, classified as perianal abscess, which occurred in the control arm and was unrelated to the study medication. There were no reports of death during the study.

## Discussion

Allergic rhinitis is an inflammatory disease of the nasal mucosa with high prevalence worldwide – which has been increasing in recent years, enhancing the interest of different medical fields and of public health in the subject.

In persistent rhinitis, nasal obstruction is often the predominant symptom. In these cases, corticosteroids are the drugs of first choice and may or may not be associated with antihistamine/decongestant,^20^ constituting the only drug class that promotes significant improvement of all symptoms, such as pruritus, sneezing, rhinorrhea, and nasal congestion.^8^

The update of the ARIA clinical recommendations^1^ following the approach taken by GRADE (grading of recommendations assessment, development, and evaluation) indicates the use of intranasal corticosteroids for the treatment of allergic rhinitis in adults and also suggests their use in children. This decision indicates the high value of the efficacy of intranasal corticosteroids and the low value of their possible adverse events.^8^

Mometasone furoate is a potent corticosteroid, as demonstrated by receptor affinity, and it has a lower bioavailability compared to other formulations.^21^ High power and low availability confer efficacy and safety to mometasone furoate.^22^

The efficacy primary analysis, performed considering the primary endpoint in the PP population, demonstrated the non-inferiority of the investigational drug in relation to the control drug for the treatment of persistent allergic rhinitis of any intensity, since the upper limit of the 95% CI for the difference between the success rates after four weeks of treatment (12.7%) was below the non-inferiority margin defined (13.7%).

Most studies on intranasal corticosteroids in patients with mild to moderate persistent allergic rhinitis identified in the literature evaluate nasal symptoms using the TNSS scale, which includes nasal obstruction, sneezing, rhinorrhea, and pruritus. These symptoms are individually ranked from 0 (no symptoms) to 3 (severe symptoms) and the total score ranges, therefore, from 0 to 12. A decrease of at least 0.55 in this score can be considered clinically significant. The present study used a scale which includes only nasal obstruction, rhinorrhea, and sneezing as symptoms, also ranked from 0 (no symptoms) to 3 (severe symptoms), ranging from 0 to 9. Thus, the use of the value 0.55 in the present study can be considered conservative.

Most individuals with allergic rhinitis have ocular symptoms – about two-thirds of asthma patients have symptoms of allergic rhinoconjunctivitis.^23^ However, the diagnosis of allergic conjunctivitis is undervalued by physicians. Ocular symptoms associated with allergic rhinitis are caused by the direct contact of the allergen with the conjunctival mucosa, and nasal-ocular reflexes would constitute an indirect pathway, depending on histamine release, since it is blocked with topical intranasal antihistamine. Evidence-based clinical research shows that intranasal corticosteroids promote relief of nasal and ocular symptoms, but the mechanism how intranasal corticosteroids improve ocular symptoms remains unknown. ARIA guidelines recommend the use of intranasal corticosteroids for treatment of allergic rhinoconjunctivitis.^8^, ^24^

Regarding security, few numerical differences were observed between groups in some of the analyses, and it seems that there is not a pattern that allows asserting which of the treatment is less toxic. Some adverse events were recorded in only one of the study groups. For adverse events that were recorded in both groups and could be compared to the frequency, no statistically significant differences between treatments were found, except for epistaxis and myalgia – more frequent in the control arm – and abnormal electrocardiogram – more common in the investigational arm. However, it is important to note that the adverse events were not related to the drugs assessed in this study. The main adverse events of intranasal corticosteroids are rare, even when the use is extended.^22^

Measurement of plasma cortisol in the morning is sensitive enough to determine systemic activity of corticosteroids on the adrenal gland, although other tests might be used with greater accuracy for this purpose.^21^ A recent review, including more than 20 studies and 6000 patients treated with mometasone furoate, found no effect on the hypothalamic-pituitary axis.^25^ Furthermore, the use of mometasone furoate for 12 months did not cause atrophy and metaplasia of the nasal mucosa, representing further evidence of its safety, even with prolonged use.^26^

Although frequently seen as a trivial, fleeting, or minor illness, allergic rhinitis can significantly impair the quality of life of patients, affecting school learning and workplace productivity.^27^, ^28^ This is because symptoms such as sneezing in bursts, nasal pruritus, and nasal congestion can lead to fatigue, difficulty in concentrating and learning, headache and, in some cases, sleep disorders such as apnea.^29^, ^30^, ^31^

## Conclusion

Mometasone furoate is a safe and effective drug for the treatment of persistent allergic rhinitis. The study demonstrated non-inferiority in efficacy and safety of investigational drug compared to the control drug, validating the proposed development as an option for the treatment of patients with mild, moderate, or severe persistent allergic rhinitis.

## Conflicts of interest

The authors declare no conflicts of interest.
